# Navigating the Crossroads of Cell Therapy and Natural Heart Regeneration

**DOI:** 10.3389/fcell.2021.674180

**Published:** 2021-05-11

**Authors:** Stefan Elde, Hanjay Wang, Y. Joseph Woo

**Affiliations:** ^1^Department of Cardiothoracic Surgery, Stanford University, Stanford, CA, United States; ^2^Stanford Cardiovascular Institute, Stanford University, Stanford, CA, United States; ^3^Department of Bioengineering, Stanford University, Stanford, CA, United States

**Keywords:** regeneration, cardiac regeneration, heart regeneration, stem cells, angiogenesis, cell therapy, cardiac, ischemic heart disease

## Abstract

Cardiovascular disease remains the leading cause of death worldwide despite significant advances in our understanding of the disease and its treatment. Consequently, the therapeutic potential of cell therapy and induction of natural myocardial regeneration have stimulated a recent surge of research and clinical trials aimed at addressing this challenge. Recent developments in the field have shed new light on the intricate relationship between inflammation and natural regeneration, an intersection that warrants further investigation.

## Introduction

Ischemic heart disease (IHD) affects more than 197 million people worldwide and accounts for the greatest number of years of life lost in the world (Virani et al., 2021). The cost of caring for these patients is expected to double over the next two decades (Heidenreich et al., 2011). Initial enthusiasm for cell therapy in IHD has been tempered by neutral clinical trial results despite signals of efficacy in animal models. Broadly, there are two cell therapy strategies being actively investigated for the potential treatment of IHD: (1) repopulating fibrotic myocardium with stem cells of various lineages, and (2) stimulating native cells of the myocardium to re-enter the cell cycle via paracrine signaling. Some of these approaches have shown potential for clinical utility, but the underwhelming translation of these therapies from animal models to patients suggest that large gaps remain in our mechanistic understanding of these processes. However, recent work has suggested an intricate relationship between inflammation, angiogenesis, and myocardial regeneration. This mechanism may explain the neutral results of cell therapy clinical trials and has sparked a new avenue for investigation that may have implications for future therapies aimed at activating natural regenerative pathways in humans.

## Stem Cell Therapy

There are two general strategies for cardiac regeneration after myocardial infarction (MI) currently under investigation. The first is direct transplantation of stem cells to the injured myocardium, which is an area of active study with many recent clinical trials (Ascheim et al., 2014; Menasché, 2018; Yau et al., 2019; He et al., 2020). The second strategy is to *redirect* resident cells of the myocardium to adopt a cardiomyocyte (CM) fate themselves. The connection between the acute inflammatory response and myocardial regeneration is an emerging area of interest.

### Stem Cell Transplantation in Animal Models

Efforts to capitalize on the regenerative potential of stem cells to repair or restore injured myocardium have been ongoing since the 1990s. After Loren Field’s group demonstrated the feasibility of grafting syngeneic CMs into recipient myocardium without rejection, first in mice (Soonpaa et al., 1994) and then in a canine model (Koh et al., 1995), there was newfound optimism for the therapeutic implications. Shortly afterward, multiple studies demonstrated the ability of rat and rabbit skeletal myoblasts to engraft in injured myocardium, adapt to the cardiac workload, and showed some potential to augment myocardial performance (Chiu et al., 1995; Murry et al., 1996; Taylor et al., 1998). Eventually, human embryonic stem cell-derived CMs were studied in a non-human primate model of MI and showed robust remuscularization of the infarcted tissue with successful electromechanical coupling (Chong et al., 2014), but with an increased rate of ventricular arrhythmias. Subsequent non-human primate studies showed similar findings of modest myocardial regeneration with 11.6% of the infarct territory remuscularized (Shiba et al., 2016; Liu et al., 2018).

### Clinical Trials

By the year 2000, trials in humans had begun. Phillippe Menasché’s team injected skeletal myoblasts into the scarred myocardium of patients undergoing coronary artery bypass grafting (CABG) in a Phase 1 trial (Menasché et al., 2001). After achieving reassuring safety endpoints, a randomized, placebo-controlled, double-blind study named the Myoblast Autologous Grafting in Ischemic Cardiomyopathy (MAGIC) trial aimed to compare high versus low dose skeletal myoblast versus placebo injections during CABG (Menasché et al., 2008). The outcomes of this study were disappointing. The myoblast groups did not show any improvement in left ventricular (LV) and ejection fraction (EF) but did result in a decrease in LV volume and an increased rate of arrhythmias (although by 6 months the rate of arrhythmias was similar between groups). While Menasché’s group reported an impressive durability of skeletal myoblasts in these trials (e.g., tissue samples from one patient showed evidence of engraftment 16 years after the initial treatment), other cell types were also being investigated.

Given the accessibility and low cost of bone marrow-derived stem cells, there was a rush to initiate clinical trials using these heterogeneous progenitors despite limited evidence that they could regenerate infarcted myocardium. Indeed, several of these trials showed no clinically significant improvement over CABG alone (Hendrikx et al., 2006; Ang et al., 2008; Pätilä et al., 2014). The reinfusion of enriched progenitor cells and infarct remodeling in acute myocardial infarction (REPAIR-AMI) trial enrolled 204 patients to investigate intracoronary infusion of bone marrow derived stem cells 3–7 days after revascularization for acute MI and showed a slight improvement in LV and EF. However, a follow up study, the bone marrow transfer to enhance ST-elevation infarct regeneration (BOOST) trial, showed that the modest improvement in LV and EF was no longer evident by 18 months, tempering optimism. Despite disappointing results in these early trials, investigation continued as proponents wondered if the results were confounded by concurrent coronary revascularization and inconsistent proportions of subtypes of mononuclear stem cells within different autologous samples (Menasché, 2018). However, the FOCUS-CCTRN trial results published in 2012 addressed one of these concerns. The FOCUS-CCTRN trial compared catheter-deliverable transendocardial bone marrow-derived stem cells to patients with chronic heart failure, and showed no improvement in LV end systolic volume, maximal oxygen consumption, defect size, or reversible wall motion abnormalities (Perin et al., 2012). While the FOCUS-CCTRN trial eliminated the confounding variable of concurrent revascularization, the issue remained that many different subtypes of mononuclear cells (MNC), mostly of unknown significance, are being delivered simultaneously which may explain seemingly discordant results between comparably designed studies.

Nonetheless, some wondered if cell therapy may still have clinical utility that is simply not reflected in multifactorial metrics such as EF, and that perhaps including more acutely ill patients in future studies would reveal a signal (Rosenzweig, 2006). In 2014, the first randomized trial of intramyocardial injection of a low dose of allogeneic mesenchymal precursor cells (MPC) in patients undergoing left ventricular assist device (LVAD) implantation showed a signal of efficacy. Patients in the MPC group had a greater probability of temporary weaning of the LVAD at 90 days than the sham group. In 2019, the Cardiothoracic Surgical Trials Network published results from a follow up trial demonstrating that injection of high dose MPCs into the failing ventricle at the time of LVAD placement did not improve the proportion of patients able to successfully wean from the LVAD over 6 months, despite a signal in the earlier phase 2 trial mentioned above (Yau et al., 2019). However, when the subset of patients with IHD were analyzed in an exploratory *post hoc* analysis, this group did show a modest benefit in temporary weaning from LVAD compared to the control group, suggesting there is a differential response among the various etiologies of heart failure. The differential effect of MPCs on patients in this trial with ischemic versus non-ischemic heart failure implies that there may be potential therapeutic benefit, perhaps via different mechanisms than initially believed.

Inconsistent results from clinical trials of stem cell transplantation may be related to ambiguity about the putative mechanism. Evidence from several studies suggest that the beneficial effects of cell therapy may be attributable to paracrine signaling induced by the transplanted progenitor cells, rather than direct repopulation of the injured myocardium (Williams and Hare, 2011; Sanganalmath and Bolli, 2013). Specifically, one study using an ovine model of non-ischemic cardiomyopathy demonstrated sparse engraftment of green fluorescent protein-labeled MPCs throughout the myocardium, but an improvement in ventricular function (Psaltis et al., 2010). Another large animal study found that when MPCs were injected into the infarct border zone at the time of acute MI, lower doses of MPCs resulted in the greatest improvement in LV remodeling and smaller infarct size relative to the control group and the high dose MPC group (Dixon et al., 2009). The results of these studies were discordant with the hypothesis that the therapeutic mechanism of cell therapy was engraftment and replacement of the non-viable CMs.

Suspecting alternative mechanisms for the findings of earlier studies on cell therapy for IHD, one of the most elegant studies to support the paracrine hypothesis came from Vagnozzi and Molkentin (Vagnozzi et al., 2020). Cellular debris from bone marrow mononuclear cells (bm-MNC) or cKIT+ cardiac progenitor cells (CPCs) were injected into the MI border zone in a mouse model. Not only did the cellular debris trigger a robust inflammatory immune response and improve fractional shortening and ventricular remodeling, but exogenous Zymosan – which activates sterile inflammation – had the same effect. In fact, the Zymosan group actually resulted in the greatest increase in CD31+ endothelial cell proliferation, compared to the injected bone marrow MNCs and CPCs. However, neither the Zymosan nor the CPCs or MNCs stimulated CM proliferation. Importantly, the improvement in ventricular function was abrogated by concurrent administration of cyclosporine, suggesting that activation of the inflammatory response after injury may underlie the potential of cell therapy rather than transdifferentiation of transplanted cells, as previously believed. Additional reports demonstrating the necessity of a sterile immune response in post-MI remodeling support this hypothesis and may partially explain the results from the human trial of MSC injections during LVAD implantation.

While Vagnozzi et al. (2020) showed that the acute inflammation triggered by cellular debris appears to modestly improve ventricular function, there was no evidence of CM proliferation. Similarly, a study examining the regenerative mechanisms in an axolotl cryoinjury model demonstrated that macrophage depletion abrogated myocardial regeneration despite cardiomyocyte proliferation (Godwin et al., 2017). Collectively, these findings suggest that the innate inflammatory response after myocardial injury is preserved across species and may result in recovery of ventricular function, but does not necessarily proceed to full scale myocardial regeneration. Additionally, applying principles from studies of regeneration in other organ systems, such as dental pulp (Marrelli et al., 2018), there is evidence to suggest that a coordinated microenvironmental remodeling response driven by the innate immune system may be essential to provide the appropriate biomechanical stimulus to drive regeneration. This informed many new lines of inquiry and will guide the design of future clinical trials.

It remains unclear whether the findings of the Vagnozzi study and those of previous clinical trials are on the mechanistic continuum of natural myocardial regeneration seen in neonatal small mammals, or if it is a non-specific antifibrotic damage-control strategy employed by the cell which is parallel to but does not result in natural regeneration, as it appears to be in the axolotl (Godwin et al., 2017). Further studies investigating the transition from the acute inflammatory-based wound-healing response to angiogenesis and myocardial regeneration are needed.

## Natural Regeneration

The distinct challenge in reprogramming mature CMs in cell cycle arrest is our relative knowledge deficit surrounding the mechanisms for releasing CM cell cycle arrest. Native CM proliferation and migration into injured territories occurs physiologically in small and large mammalian neonates (Ingason et al., 2018; Ye et al., 2018; Das et al., 2019; Wang et al., 2020a, b). However, attempts to reactivate this pathway in adult mammals have proven challenging.

### Hypoxia and Angiogenesis

Although the adult mammalian heart is unable to regenerate to any significant extent, neonatal mammals have the capacity to regenerate viable myocardium following MI in the early days of life (Haubner et al., 2012, 2016; Porrello et al., 2013; Wang et al., 2020a, b). It follows to reason that angiogenesis must precede myocardial regeneration. This is driven by the development of a network of collateral arteries formed by arterial endothelial cells (Ingason et al., 2018; Das et al., 2019), which are able to develop into arteries (Simons and Eichmann, 2015).

Efforts to harness the natural regenerative capacity have focused on releasing CMs from cell cycle arrest via a variety of approaches. Releasing cell cycle arrest has been attempted with overactivation of cell cycle activators such as cyclin D2, which led to enhanced angiogenesis and remuscularization in the MI border zone (Zhu et al., 2018). Similarly, the activation of Yap – a downstream effector of the Hippo pathway, involved in vascular remodeling and angiogenesis – has been shown to extend cell cycle activity and CM proliferation (Lin and Pu, 2014). Small molecules such as neuregulin also appear to promote CM proliferation in mice and in humans (Polizzotti et al., 2015), leading to improved ventricular function and reduced scar size after MI in small and large animal models (Cohen et al., 2014, 2020). It also appears possible to reprogram resident cardiac fibroblasts to adopt a CM fate by using small molecule inhibitors of transforming growth factor beta and WNT, which resulted in successful transdifferentiation and improved ventricular function in mice (Mohamed et al., 2017). Interestingly, zebrafish retain regenerative capacity throughout their life and have a relatively hypoxic circulatory system as a result of veno-arterial mixing in their two-chamber heart, similar to the shunt dependent phase of mammalian gestation (Cardoso et al., 2020). This hypoxic environment drives anaerobic glycolysis, the predominant energy source in the prenatal heart. However, upon rapidly transitioning to higher postnatal oxygen content levels, a metabolic transition to oxidative metabolism occurs. As the levels of glycolytic enzymes fall over the first postnatal week, those that are involved in oxidative metabolism increase reciprocally. The reactive oxygen species produced by the sudden spike in mitochondrial respiration induce damage to proteins, lipids, and DNA, which stimulates the DNA damage response that ultimately leads to cell cycle arrest and polyploidization (Cardoso et al., 2020). The sudden oxidative stress and collateral damage that leads to cell cycle arrest appears to occur over the same timespan that neonatal mammalian hearts lose their regenerative capacity, and scavenging of reactive oxygen species appears to prolong the proliferative phase of the mammalian myocardium (Puente et al., 2014).

As evidence mounted that the findings in the cell therapy clinical trials were more likely related to paracrine signaling induced by transplanted cells rather than engraftment, some groups began attempting to recapitulate the responsible signaling pathways without the technical challenges of engrafting billions of cells. After MI, the acute inflammatory response attracts macrophages, fibroblasts, and T cells to the infarct zone. These cells clear debris from apoptotic cells and remodel the extracellular matrix. In turn, potent chemoattractants such as stromal cell-derived factor 1α (SDF) recruit endothelial progenitor cells to the infarct territory and promote angiogenesis (Frederick et al., 2010). By using advanced computer modeling, Hiesinger et al. (2011) developed an engineered SDF analog (ESA) which demonstrated improved efficiency in stimulating microrevascularization in an ischemic cardiomyopathy mouse model, which was then subsequently validated in a rat hindlimb model and ovine MI model (Hiesinger et al., 2011; Macarthur et al., 2014; Edwards et al., 2016). Many groups began attempting acellular delivery of extracellular vesicles (EVs) loaded with signaling molecules to the injured myocardium, activating the inflammatory and possibly regenerative responses. The cellular response to their cargo of proteins, lipids, and micro-RNAs (miRNAs) activates a variety of pathways depending on the specific composition of the EV cargo. EVs can also be enriched with pre-selected small molecules, proteins, or miRNA to modulate the intended response from the host cell, thus serving as an attractive vehicle to influence cell fate (de Abreu et al., 2020). In a porcine model of acute MI, human exosomes delivered intramyocardially decreased infarct size by about 20% and mildly improved LV remodeling (Gallet et al., 2017). While these approaches appear to hold potential to attenuate scar formation, the path toward a clinical trial with EVs most likely will involve rigorous purification, laboratory validation, and standardization testing, which is likely to be an expensive and lengthy process due to safety concerns regarding off-target effects.

**FIGURE 1:**
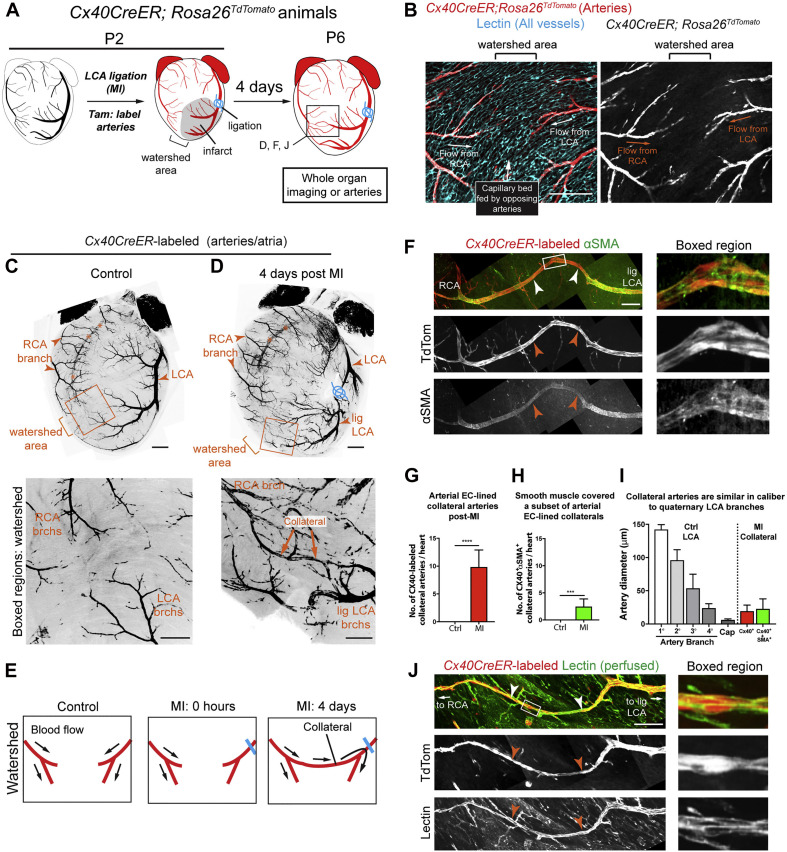
Extensive Collateral Artery Formation in the Neonatal Mouse Heart 4 days after MI. **(A)** Experimental design where *Cx40CreER*-labeled arteries are shown in red. **(B)** Confocal image of a watershed area from a P2 control heart. The capillary bed (cyan) is fed by left coronary artery (LCA) and right coronary artery (RCA). **(C,D)** Confocal images (anterior views) of control **(C)** and myocardial infarction (MI) **(D)** hearts. Arterial ECs are shown in black. MI induced collateral arteries that connect ligated (lig) branches (brchs) of the LCA with RCA branches across the watershed area (boxed regions). Asterisks indicate the RCA showing through from the posterior heart wall. **(E)** Schematic of how collateral arteries restore blood flow (arrows) to injured myocardium. Arteries, red; ligation, blue. **(F)** Some *Cx40CreER*-labeled collateral arteries contain smooth muscle (white and red arrows). **(G,H)** Quantification of *Cx40CreER*-labeled **(G)** and smooth muscle-covered **(H)** collateral arteries. Hearts: *n* = 8 control, *n* = 12 MI. **(I)** Collateral arteries were similar in diameter to quaternary (4°) branches. Arteries: *n* = 24 Cx40^+^, *n* = 9 αSMA^+^. **(J)** Collateral arteries were perfused (white and red arrows). Cap, capillaries; P, postnatal; Tam, tamoxifen; EC, endothelial cells; TdTom, tdTomato. Scale bars: **(B)**, 200 μm; **(C,D)** low magnification, 500 μm; **(C,D)** boxed region, 200 μm; **(F,J)**, 200 μm. Error bars indicate SD. ^***^p ≤ 0.001, ^****^p ≤ 0.0001. Adopted from Das et al. (2019).

Interpreting the Vagnozzi et al. (2020) study in this context suggests that a possible link between natural regeneration and cell therapy is the inciting inflammatory immune response. This is reinforced by recent work demonstrating that arterial endothelial cells in adult mice can be induced to undergo artery reassembly (i.e., neoangiogenesis) with the administration of exogenous SDF (Das et al., 2019; Figure 1). Exogenous administration of SDF or engineered analogs such as ESA have also been shown to augment ventricular function, reduce scar size, and restore myocardial biomechanics in both small and large animal models (Hiesinger et al., 2011; Macarthur et al., 2014; Wang et al., 2019). This is notable because SDF and its receptor CXCR4 have well-documented roles in immune activation and are upregulated by inflammatory cytokines such as VGEF, TGFB1, and bFGF (García-Cuesta et al., 2019). Therefore, it appears plausible that we are getting closer to elucidating the transition between the acute inflammatory response and natural regeneration.

Unsurprisingly, excessive proliferation or inappropriate depletion of resident cardiac macrophages after acute MI can impair myocardial regeneration in mice (Aurora et al., 2014; Lavine et al., 2014; Dick et al., 2019) and result in an abnormal remodeling response. Specifically, macrophages in P1 mice seem to secrete soluble factors that stimulate angiogenesis (Aurora et al., 2014). In addition to macrophages, T-regulatory cells are also necessary for CM proliferation and myocardial regeneration (Li et al., 2019). Conversely, ablation of specific subsets of CD4+ T-cells actually improves cardiomyocyte proliferation and reduces fibrosis by directing macrophages away from the M2 phenotype (Bansal et al., 2019; Li et al., 2020). Neutrophils have traditionally been considered exclusively pro-inflammatory agents in the response to myocardial injury. However, neutrophil depletion studies have showed impaired ventricular functional recovery and an uncoordinated fibrotic response after MI (Puhl and Steffens, 2019). Neutrophils have been shown to play a role in the clearance of cellular debris and recruitment and activation of specific M2 macrophage subsets that promote clearance of apoptotic cells via neutrophil gelatinase-associated lipocalin (Horckmans et al., 2017). Given the importance of preserving the mechanical properties of native myocardium in recovery of ventricular function after MI (Wang et al., 2019), and to optimize the bioscaffold in which progenitor cells may adhere (Ballini et al., 2017), it is plausible that an uncoordinated inflammatory or fibrotic response may result in an inadequate microenvironment for cardiomyocyte regeneration. Additionally, the complement pathway receptor 5a is evolutionarily conserved and promoted CM proliferation in a recent cross-species study (Natarajan et al., 2018; Vujic et al., 2020). The growing evidence for the role of inflammation and paracrine signaling recalibrated the focus of investigators toward endogenous pathways that regulate the stepwise progression from infarct maturation and innate wound healing to angiogenesis and subsequent myocardial regeneration.

## Conclusion and Future Directions

While this review focuses on cell therapy and paracrine signaling approaches to myocardial regeneration, innovative approaches in other fields such as hydrogels, biophysics, neurohormonal modulation, and tissue engineering have also yielded promising advancements toward the common goal of myocardial regeneration. The exponential pace of discovery of new genes, small molecules, and proteins that promote various steps of myocardial regeneration has identified many promising avenues of research. The neutral results of clinical trials investigating cell therapy for IHD may be partially explained by the emergence of an alternative inflammatory mechanism underlying the rescue of ventricular function after MI. It appears that the small therapeutic benefit of cell therapy is more likely related to endogenous inflammatory and wound-healing pathways, rather than direct replacement of the damaged myocardium with viable cardiomyocytes. This is a significant paradigm shift in the pursuit of myocardial regeneration after injury. Therapies targeting individual stimuli for regeneration have shown potential for success but have fallen short of the robust physiologic regenerative response seen in neonatal mammals. Nonetheless, repopulating the infarcted myocardium with viable, non-arrhythmogenic CMs remains the ultimate goal, and recent work in animal models on natural regeneration remains a promising field for further investigation. Future progress will likely depend upon interdisciplinary collaborations, including bioengineers, immunologists, cell biologists, and physicians to develop multifaceted therapeutic strategies that integrate bioengineering and developmental biology techniques.

## Author Contributions

YJW and HW conceptualized the manuscript which was written by SE, and edited and revised the manuscript. All authors contributed to the article and approved the submitted version.

## Conflict of Interest

The authors declare that the research was conducted in the absence of any commercial or financial relationships that could be construed as a potential conflict of interest.
